# Human Alveolar Macrophages Detect SARS‐CoV‐2 Envelope Protein Through TLR2 and TLR4 and Secrete Cytokines in Response

**DOI:** 10.1111/imm.13922

**Published:** 2025-05-04

**Authors:** Conor Grant, Emily Duffin, Finbarr O’Connell, Parthiban Nadarajan, Colm Bergin, Joseph Keane, Mary P. O'Sullivan

**Affiliations:** ^1^ Department of Clinical Medicine Trinity Translational Medicine Institute, St. James's Hospital, Trinity College Dublin, the University of Dublin Dublin Ireland; ^2^ Department of Infectious Diseases Trinity Translational Medicine Institute, St. James's Hospital, Trinity College Dublin, the University of Dublin Dublin Ireland

**Keywords:** age, COVID‐19, macrophage, risk factors, SARS‐CoV‐2, smoking

## Abstract

Alveolar macrophages (AMs) are the most numerous immune cells of the lung and are the resident, sentinel lung immunocytes that summon trafficking immune cells to the compartment. Immune profiling of AMs from COVID‐19 patients implicates AMs in the immune circuits that drive pulmonary inflammation in severe COVID‐19 infection. However, little is known about human AM responses to SARS‐CoV‐2 proteins, such as the spike protein and envelope protein. We aimed to understand if human AMs recognize SARS‐CoV‐2 proteins and how they respond. We found that human AMs do not sense SARS‐CoV‐2 spike protein but do sense envelope protein via the pattern recognition receptors TLR2 and TLR4, secreting IL‐1β, IFNγ, IL‐12p70, IL‐6, and TNFα in response. AMs from donors over the age of 70 years produced significantly more cytokines than those from younger patients following stimulation with SARS‐CoV‐2 envelope protein. AMs from current smokers had lower cytokine secretion. This is the first report of human AMs producing cytokines in response to SARS‐CoV‐2 proteins and the first to correlate those responses with clinical risk factors. These results may partly explain why older adults are at such high risk of severe lung inflammation in COVID‐19.

## Graphical abstract

Alveolar macrophages (AMs)—the most numerous immune cells of the lung—respond to viral infections by secreting cytokines to summon other immune cells. We investigated whether AMs recognize SARS‐CoV‐2 proteins and how they respond. AMs did not sense spike protein but did recognize envelope protein via the pattern recognition receptors TLR2 and TLR4, secreting pro‐inflammatory cytokines in response. AMs from donors over the age of 70 produced more cytokines than those from younger adults following stimulation with envelope protein. These results may partly explain why older adults are at such high risk of severe lung inflammation in COVID‐19.

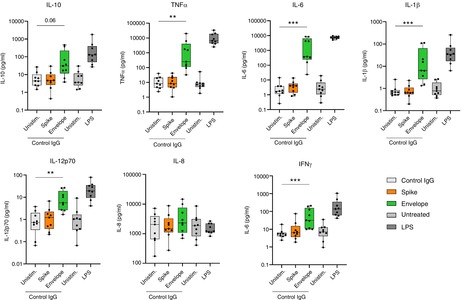

## 1 Introduction

Severe COVID‐19 is characterized by interstitial pneumonitis, acute respiratory distress syndrome (ARDS) and a high associated mortality. Alveolar macrophages (AMs) are the most numerous immune cells of the lung and are its resident, sentinel immunocytes that summon other innate and adaptive cells and instruct their activation. AMs are known to be important in the host's defence against viral infections such as influenza A [1, 2]. It is likely that AMs are the first professional immune cells to encounter SARS‐CoV‐2 following transmission. Because AMs can exert their influence so early in the infection, before any homing monocytes, neutrophils, or T cells arrive in the alveoli, they may play an outsized role in determining the subsequent disease course. Indeed, single‐cell RNA sequencing of the alveolar compartment in COVID‐19 patients supports the centrality of AMs in the immune circuits that drive pulmonary inflammation in severe disease [3, 4]—SARS‐CoV‐2 infected AMs have increased expression of cytokines and chemokines including IL‐1β, CCL2,and CXCL10 and recruit memory T cells to the lung. The T cells secrete IFNγ, activating the infected AMs, with subsequent death of these SARS‐CoV‐2 infected AMs, which was associated with immunopathology in the lung [4].

Despite the intriguing possibility of AMs' influence on the clinical trajectory in COVID‐19, they are relatively understudied. The weight of the evidence so far suggests that human AMs are not supportive of SARS‐CoV‐2 replication [5, 6, 7]. Nevertheless, viral antigens and RNA have been detected in AMs from COVID‐19 patients [4, 8] and envelope protein is present in the serum of infected patients [9]. Murine studies found that the depletion of AMs was associated with reduced lung pathology following SARS‐CoV‐2 infection in vivo [10]. Therefore, while human AMs are unlikely to support SARS‐CoV‐2 replication, there are indications that AMs may play an important role in the pathophysiology of severe COVID‐19, perhaps through the production of cytokines in response to viral components. There are no experimental data to indicate whether AMs secrete cytokines following contact with the SARS‐CoV‐2 virus. AMs express surface pattern recognition receptors (PRRs) including TLR4, which may detect SARS‐CoV‐2 antigens such as spike protein or envelope protein. Multiple studies have investigated whether these SARS‐CoV‐2 antigens engage TLR2 and TLR4 in macrophages and have produced variable and sometimes conflicting results [11, 12, 13, 14, 15, 16, 17, 18]. Importantly, none of these studies examined the sentinel, resident immune cell of the lung—the human AM.

Advanced age is the strongest clinical risk factor for poor outcomes in COVID‐19. Booth et al. collated the clinical data from over 17 million patients with COVID‐19 and found that being over 75 years of age conferred an adjusted odds ratio (OR) of 1.93 for severity and 5.82 for mortality from COVID‐19. These risks were considerably higher than for other well‐documented risk factors such as cardiovascular disease and obesity [19]. These worse outcomes in the elderly are associated with an increased risk of pulmonary hyperinflammation and ARDS [20]. Another clinical variable—smoking—not only impairs pulmonary function over time but has been associated with an increased risk of hospitalisation and death from influenza [21, 22]. It is perhaps surprising, therefore, that Booth et al. found active smoking to confer quite modest risks in COVID‐19 [19].

We aimed to understand if human AMs recognize SARS‐CoV‐2 spike and envelope proteins, and if so by what surface PRR and what cytokines are produced as a result. We also aimed to discover if the cytokine responses of human AMs to these antigens vary according to the clinical risk factors of age and smoking status—a possible mechanism underlying the different disease trajectories followed by these patient cohorts.

## 2 Methods

### 2.1 Cell Culture of Human AMs

Human AMs were retrieved from bronchoalveolar lavage (BAL) fluid of consenting patients undergoing routine bronchoscopy at St. James's Hospital, Dublin. Ethics was approved by St. James's Hospital/Tallaght University Hospital Joint Research Ethics Committee. Exclusion criteria for collection included a diagnosis of lung cancer, sarcoidosis, HIV, or any active infection. BAL fluid was filtered through a 100 μm nylon strainer (BD Falcon; BD Bioscience, Erembodegem, Belgium).

The AMs were pelleted by centrifugation at 150 g for 15 min, resuspended at 2.5 × 10^5^ cells/mL in RPMI (ThermoFisher) supplemented with 10% foetal calf serum (Sigma‐Aldrich), 2.5 μg/mL Fungizone and 5 μg/mL cefotaxime (Invitrogen) and 1 mL/well was added to untreated 48‐well plates (ThermoFisher). AMs were incubated at 37°C, 5% CO_2_ for 24 h and then washed to remove non‐adherent cells, replacing it with fresh media before experimentation.

### 2.2 Treatment of Macrophages With Antibodies

Monoclonal anti‐human TLR2 IgG2a kappa (anti‐TLR2) (ThermoFisher; 16‐9922), monoclonal anti‐human TLR4 IgG2a kappa (anti‐TLR4) (ThermoFisher; 16‐9917) and isotype control anti‐mouse IgG2a kappa (ThermoFisher; 16‐4724) were reconstituted in sterile PBS and stored at 4°C prior to experimentation. The cells were pre‐treated with 10 μg/mL of anti‐TLR2, anti‐TLR4,or the isotype control antibody for 1 h prior to stimulation with envelope or spike protein or TLR ligands.

### 2.3 Stimulation of Macrophages With TLR Ligands

The TLR4 ligand lipopolysaccharide (LPS) (Sigma‐Aldrich; L5418) was used at a concentration of 100 ng/mL. The TLR2/1 ligand Pam3CSK4 (Invivogen; tlrl‐pms) was used at concentrations of 10 and 100 ng/mL.

### 2.4 Stimulation of Macrophages With SARS‐CoV‐2 Antigens

The AMs were treated for 1 h with blocking antibodies against TLR2 or TLR4 or an isotype control antibody as described above and were then stimulated with SARS‐CoV‐2 spike protein (R&D; 10 549‐CV; < 0.1 EU/μg by LAL method) at 1 μg/mL, envelope protein (ABclonal; RP01263; < 0.1 EU/μg by LAL method) at 1 μg/mL, or left unstimulated. An untreated, LPS‐stimulated (100 ng/mL) condition was used as a positive control. Supernatant was collected at 24‐h poststimulation, and the concentration of cytokines was measured using a Meso Scale Discovery (MSD) multiplex assay (Human Proinflammatory 7 spot plate, N75008B‐1 or Human Proinflammatory‐ 4 spot plate, N45009B‐1) according to the manufacturer's instructions (Meso Scale Diagnostics, Rockville, Maryland, USA). The plate was read on a MESO Quick Plex SQ 120 instrument, and the results were analysed using MSD Discovery 4.0 Workbench Analysis software.

### 2.5 Proteinase K Digestion of Envelope Protein

Proteinase K (A3830, AppliChem GmbH, Darmstad) was reconstituted in endotoxin‐free water (Merck) and was added to envelope protein (10 μg/mL) or LPS (1 μg/mL) in HBSS at a final concentration of 10 μg/mL. An equal volume of endotoxin‐free water alone was added to parallel aliquots of envelope protein and LPS as controls. Samples were incubated for 1 h at 37°C. PMSF (10 μM) was added to inhibit serine protease activity, and samples were incubated on ice for 1 h [23]. Samples were diluted in cell culture medium, and then control or Proteinase K‐treated Envelope protein (1 μg/mL) or LPS (100 ng/mL) were added to duplicate wells of AMs in a 96‐well plate. The plate was incubated for 24 h in a CO_2_ incubator at 37°C. Cytokine secretion (TNFα, IL‐6, IL‐1β, and IFN‐γ) was measured in supernatants using a 4‐plex pro‐inflammatory‐1 kit (MSD).

### 2.6 Cell Viability

Cell death was measured using the propidium iodide exclusion assay as previously described [24]. Briefly, macrophages were untreated or incubated in the presence of envelope protein for 24 h, followed by the addition of propidium iodide (5 μg/mL) to detect cells with permeabilized cell membranes, and the nuclei were counterstained with a combination of 50 μg/mL Hoescht 33 342 (Sigma B2261) and 20 μg/mL Hoescht 33 258 (Sigma B1155). Images were obtained using a Cytell Cell Imaging Analyser (GE Healthcare) and the percentage of dead (PI positive) cells was determined using the Cell Viability Bioapp.

### 2.7 Statistical Analysis

All data were analysed, and graphs were generated using GraphPad Prism software (version 9.5.0) (GraphPad Software LLC.). Statistically significant differences between groups were determined using a one‐way ANOVA, or a two‐way ANOVA if more than one variable within each group was being compared, using repeated measures and corrections for multiple comparisons where appropriate. Results are expressed as mean ± standard error of the mean (SEM). A *p* value < 0.05 was considered statistically significant. In each figure, asterisks indicate the level of statistical significance: * *p* ≤ 0.05, ** *p* ≤ 0.01, ***p ≤ 0.001.

## 3 Results

### 3.1 SARS‐CoV‐2 Envelope Protein, but not Spike Protein, Induces the Secretion of Cytokines From Human AMs

We first aimed to establish whether the SARS‐CoV‐2 spike protein or envelope protein induced a cytokine response from human AMs (Table 1). The AMs were treated with an isotype control antibody (control IgG) (10 μg/mL) and then stimulated with SARS‐CoV‐2 spike protein or envelope protein (both 1 μg/mL) or were unstimulated. Untreated AMs that were stimulated with LPS (100 ng/mL) or were unstimulated were used as positive and negative controls, respectively. Cytokine concentrations were measured in supernatant that was collected 24 h following stimulation.

**TABLE 1:** AM Donor characteristics.

Donor#	Sex	Age (years)	Smoking status	Figures 1, 2, 3, 4, 5, 6	Figure S1	Figure S2	Figure S3
1	M	70.2	Smoker	X			
2	M	43.7	Smoker	X			
3	M	79.9	Ex	X			
4	F	76.0	Smoker	X			
5	F	42.1	Ex	X			
6	F	49.3	Smoker	X			
7	F	31.9	Never	X			
8	F	85.0	Ex	X			
9	M	55.0	Smoker		X		
10	F	63.6	Smoker		X		
11	F	42.7	Ex		X		
12	F	48.4	Smoker	X	X	X	
13	F	69.3	Smoker		X	X	X
14	F	50.4	Ex			X	

**FIGURE 1:**
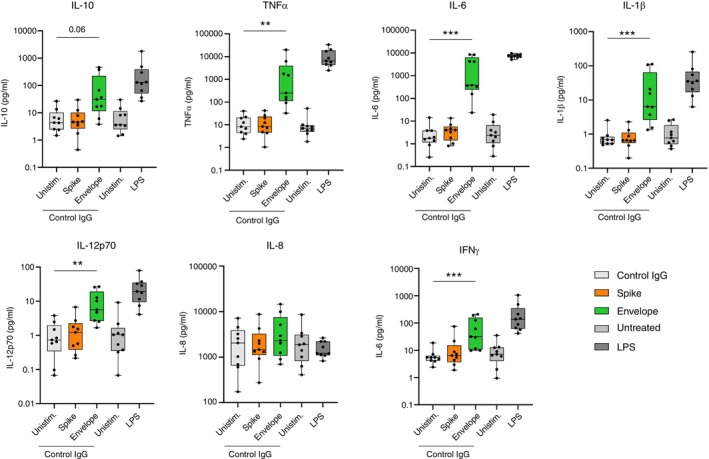
SARS‐CoV‐2 envelope protein, but not spike protein, induces the secretion of cytokines from human alveolar macrophages. AMs were isolated from BAL fluid of patients undergoing routine bronchoscopy by adherence purification. AMs were pre‐treated with an isotype control antibody (control IgG) (10 μg/mL) or untreated for 1 h and then stimulated with SARS‐CoV‐2 spike protein or envelope protein (both 1 μg/mL). Untreated AMs that were stimulated with LPS (100 ng/mL) or unstimulated were used as positive and negative controls, respectively. Supernatant was collected at 24 h poststimulation. The concentration of cytokines was measured using an MSD multiplex assay (*n* = 9). Statistical comparisons were made using the Friedman test with Dunn's multiple comparisons test.

The two unstimulated negative control conditions (untreated and control IgG treated) had statistically equivalent concentrations of all cytokines. SARS‐CoV‐2 spike protein did not induce the secretion of any of the tested cytokines above the levels of the negative controls. However, stimulation of human AMs with SARS‐CoV‐2 envelope protein resulted in a significant secretion of IL‐10, IFNγ, IL‐12p70, IL‐1β, IL‐6,and TNFα (Figure 1). IL‐8 was secreted at relatively high levels by unstimulated AMs but was not significantly induced above this baseline level by either envelope protein or, similarly to our previously published data [25], by LPS. Importantly, envelope protein did not affect AM viability (Figure S1), and proteinase K abrogated this response to envelope protein (Figure S2). From this,we conclude that human AMs detect and mount a cytokine response to the SARS‐CoV‐2 envelope protein, but not the spike protein.

### 3.2 TLR4 and TLR2 Recognize SARS‐CoV‐2 Envelope Protein on Human AMs

We next sought to understand if the SARS‐CoV‐2 envelope protein was detected by either TLR2 or TLR4. To confirm that the TLR neutralising antibodies blocked TLR signalling, human AMs were stimulated with known ligands for TLR2 (PAM_3_CSK_4_ at 10 or 100 ng/mL) or TLR4 (LPS at 100 ng/mL). PAM_3_CSK_4_ (100 ng/mL) induced the secretion of TNFα and IL‐6, which was reduced in the presence of anti‐TLR2. LPS induced IFNγ, IL‐12p70, IL‐6, and TNFα secretion from AMs, which was reduced in the presence of anti‐TLR4 (Figure S3).

**FIGURE 2:**
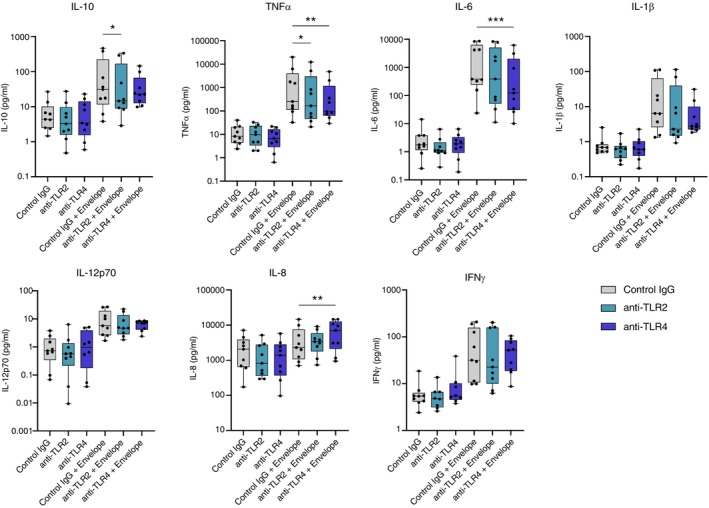
Blocking antibodies against TLR2 and TLR4 alter the cytokine responses of human alveolar macrophages to SARS‐CoV‐2 envelope protein. AMs were isolated and treated with blocking antibodies against TLR2, TLR4, or an isotype control antibody (control IgG) (all 10 μg/mL) and stimulated as previously described. Supernatant was collected at 24‐h post stimulation. The concentration of cytokines was measured using an MSD multiplex assay (*n* = 9). Statistical comparisons were made using the Friedman test with Dunn's multiple comparisons test.

Human AMs (Table 1) were treated with neutralising antibodies to TLR2 or TLR4, or an isotype control antibody (control IgG) (all 10 μg/mL) for 1 h before stimulation with SARS‐CoV‐2 envelope protein. No significant difference in the secretion of any cytokine measured was observed between the unstimulated controls in the presence or absence of the anti‐TLR antibodies or the isotype control. Blocking TLR2 significantly reduced the secretion of TNFα and IL‐10. Blocking TLR4 resulted in a significant reduction in both IL‐6 and TNFα secretion from human AMs, with a non‐significant trend towards decreased secretion of IFNγ, IL‐10, IL12p70, and IL‐1β also evident. While secreted IL‐8 levels were not significantly higher than control in envelope‐stimulated AMs, there was a significant increase in the presence of the TLR4 antibody. We concluded from this data that SARS‐CoV‐2 envelope protein is recognized by TLR2 and TLR4 on human AMs (Figure 2).

### 3.3 AMs From Older Donors Have Higher Cytokine Responses to SARS‐CoV‐2 Envelope Protein Than AMs From Younger Donors

**FIGURE 3:**
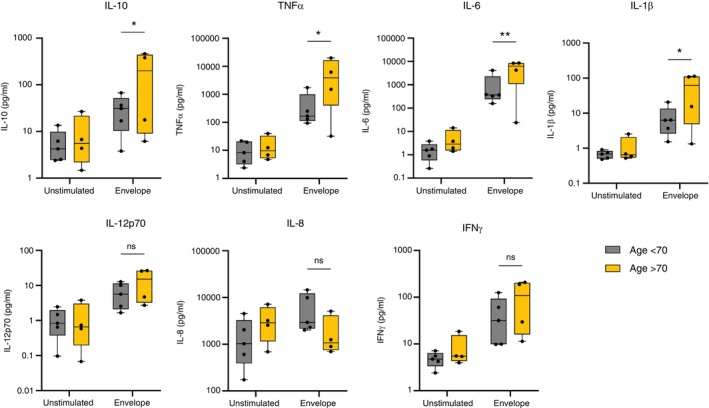
Alveolar macrophages from donors aged over 70 years had higher cytokine responses to SARS‐CoV‐2 envelope protein than younger donors. AMs were isolated, treated,and stimulated as previously described. Supernatant was collected at 24 h poststimulation. The concentration of cytokines was measured using an MSD multiplex assay. The statistical comparison presented here is younger (*n* = 5) and older (*n* = 4) donor AMs treated with control IgG, then stimulated with envelope protein, or unstimulated. Planned statistical comparisons were made between stimulated cells from donors aged < 70 years versus those from donors aged > 70 years using two‐way ANOVA with Šídák's multiple comparison test.

Those older than 70 years of age are at far higher risk of severe disease and mortality due to COVID‐19 [19]. We compared the cytokine secretion of AMs responding to envelope protein from donors older (mean 77.8 (± SEM 3.1) years) or younger (mean 43.1 (± SEM 3.1) years) than 70 years. For unstimulated AMs, there was no difference between younger and older donors for any cytokine—older and younger donor's AMs were indistinguishable at rest. Stimulation of the AMs with SARS‐CoV‐2 envelope protein resulted in a significantly higher concentration of IL‐6, IL‐10, IL‐1β, and TNFα in supernatants from donors over the age of 70 compared with those under 70 years old (Figure 3). From this we conclude that older donors' AMs are more responsive to SARS‐Cov‐2 envelope protein than younger donors AMs, producing more pro‐inflammatory cytokines.

### 3.4 AMs From Older Donors do not Have Significantly Different Cytokine Responses to LPS Than AMs From Younger Donors

**FIGURE 4:**
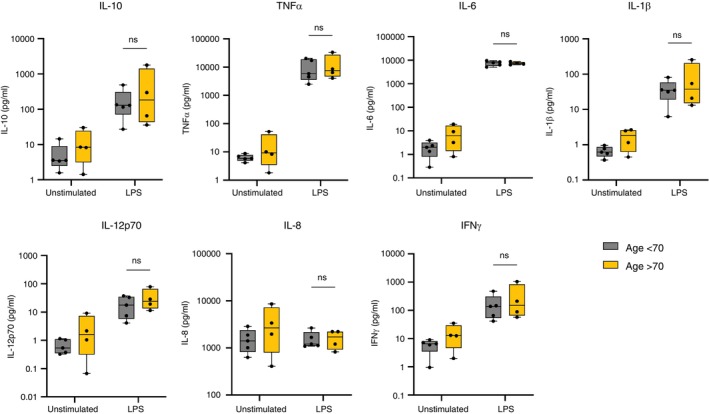
Alveolar macrophages from donors aged over 70 years did not have higher cytokine responses to LPS than younger donors. AMs were isolated, treated, and stimulated as previously described. Supernatant was collected at 24 h poststimulation. The concentration of cytokines was measured using an MSD multiplex assay. The statistical comparison presented here is younger (*n* = 5) and older (*n* = 4) donor untreated AMs stimulated with LPS or unstimulated. Planned statistical comparisons were made between stimulated cells from donors aged < 70 years versus those from donors aged > 70 years using two‐way ANOVA with Šídák's multiple comparison test.

We also compared the cytokine secretion of AMs responding to LPS from donors older or younger than 70 years old. LPS stimulation did not result in a significantly different secretion of any cytokines between younger and older donors. IL‐6 in particular, which has been independently associated with COVID‐19 severity and mortality, had nearly identical secretion by younger and older donors' AMs in response to LPS [7], whereas we observed a significant age difference following stimulation of human AMs with the envelope protein (Figure 4). This suggests that the age differences observed with the envelope protein might be a specific effect rather than a general effect of AM stimulation.

### 3.5 AMs From Smokers had Lower Cytokine Responses to SARS‐CoV‐2 Envelope Protein Than AMs From Never‐ or Ex‐Smokers

**FIGURE 5:**
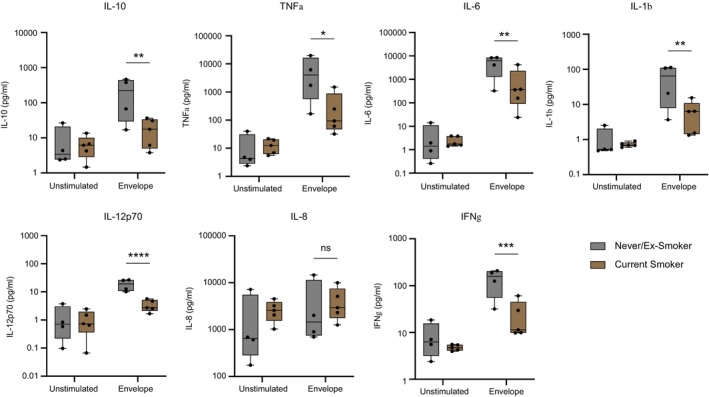
Alveolar macrophages from smokers had lower cytokine responses to SARS‐CoV‐2 envelope protein than non‐smokers. AMs were isolated, treated, and stimulated as previously described. Supernatant was collected at 24‐h poststimulation. The concentration of cytokines was measured using an MSD multiplex assay. The statistical comparison presented here is smokers' (*n* = 5) and non‐smokers' (*n* = 4) AMs treated with control IgG, then stimulated with envelope protein, or unstimulated. Planned statistical comparisons were made between stimulated cells from never/ex‐smokers versus those from current smokers using two‐way ANOVA with Šídák's multiple comparison test.

Our laboratory has previously reported impaired TNFα, IFNγ and IL‐1β secretion in smokers' AMs responding to *Mycobacterium tuberculosis* (Mtb) [25]. We wondered whether the same impairment could be observed in smokers' AMs responding to the SARS‐CoV‐2 envelope protein. Following stimulation with envelope protein, AMs from current smokers produced significantly less IFNγ, IL‐10, IL‐12p70, IL‐1β, TNFα, and IL‐6 than AMs from never‐ or ex‐smokers. Importantly, current smokers did not differ significantly in age from never‐ or ex‐smokers, so this result cannot be due to a younger mean age of smokers (mean age 59.7 vs. 57.5 years) (Figure 5). This finding aligns well with previous research indicating that smoking impairs human AMs' cytokine responses in infection [25].

### 3.6 AMs From Smokers did not Have Significantly Different Cytokine Responses to LPS Than AMs From Never‐ or Ex‐Smokers

**FIGURE 6:**
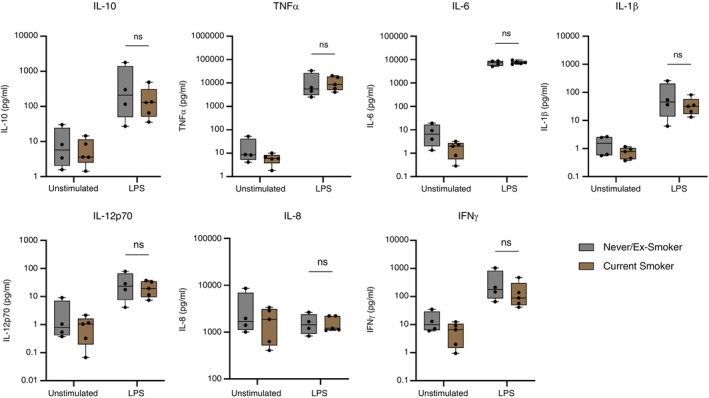
Alveolar macrophages from smokers did not have lower cytokine responses to LPS than non‐smokers. AMs were isolated, treated, and stimulated as previously described. Supernatant was collected at 24 h poststimulation. The concentration of cytokines was measured using an MSD multiplex assay. The statistical comparison presented here is smokers' (*n* = 5) and nonsmokers' (*n* = 4) untreated AMs stimulated with LPS, or unstimulated. Planned statistical comparisons were made between stimulated cells from never/ex‐smokers versus those from current smokers using two‐way ANOVA with Šídák's multiple comparison test.

We investigated if smoking also impaired human AM cytokine responses to LPS. Interestingly, as was also true for age, current smoking did not significantly affect human AM cytokine responses to LPS (Figure 6). Other researchers have found that LPS stimulation results in diminished cytokine secretion from murine and human AMs exposed to cigarette smoke [26, 27, 28]. Our findings with LPS could support the hypothesis that the effect of envelope protein we observed is somewhat specific.

## 4 Discussion

We found that SARS‐CoV‐2 envelope protein is recognized by TLR2 and TLR4 on human AMs, leading to the production of proinflammatory cytokines. AMs from older donors had higher cytokine secretion, and AMs from current smokers had lower cytokine secretion. This is the first report of human AMs producing cytokines in response to a SARS‐CoV‐2 antigen, and the first to correlate those responses with clinical risk factors. This is a newly uncovered mechanism through which innate pulmonary responses to COVID‐19 in the elderly differ from younger adults in a manner that could plausibly be pathological.

Envelope protein induced the secretion of the cytokines IL‐6, TNFα, IL‐1β, IFNγ and IL‐12p70. These cytokines have wide‐ranging effects on other innate and adaptive immune cells, including neutrophil recruitment and acute phase reactant production (IL‐6), cell death (TNFα), activation of monocytes and neutrophils (IL‐1β), activation of macrophages (IFNγ) and the differentiation of T helper cells to a Th1 phenotype (IL‐12p70) [29]. IL‐6 and TNFα are independently associated with severity and mortality in COVID‐19 [7]. However, the secretion of IL‐6 and TNFα from monocytes infected with SARS‐CoV‐2 in vitro was entirely dependent on IL‐1β [30]. IL‐1β has been detected in BAL fluid and pathological specimens from COVID‐19 patients [3, 31]. Unlike the other cytokines we measured, IL‐1β requires inflammasome activation for its own activation and secretion. Unusually, in addition to its triggering of “Signal 1” of the NLRP3 inflammasome via TLR4—SARS‐CoV‐2 envelope protein may also act as “Signal 2” through its function as a cation channel [32, 33, 34, 35, 36].

Blocking antibody against TLR4 significantly reduced the secretion of IL‐6 and TNFα, and TLR2 antibody reduced secretion of TNFα and IL‐10 from envelope protein‐stimulated AMs, compared with isotype control antibody‐treated AMs. This suggests that SARS‐CoV‐2 envelope protein is sensed by TLR2 and TLR4 on human AMs. Other researchers have also found that SARS‐CoV‐2 envelope protein signals through TLR4 in THP‐1 and murine RAW macrophages [16], and through TLR2 and TLR4 in airway epithelial cells [37] and HEK293 reporter cells [38]. Human TLR2 has been shown to interact directly with envelope protein from SARS‐CoV‐2 [15]. How envelope protein might activate TLR4 is unclear. The hydrophobic Lipid A moiety of LPS binds to MD2, leading to dimerisation of an LPS/MD2/TLR4 complex to stimulate downstream signalling pathways. Viral proteins known to activate TLR4 include VSV G protein, RSV F protein, Dengue virus NS1, and Ebola virus glycoprotein. It is not known how these proteins interact with MD2/TLR4, but they have several things in common, including being membrane‐bound, glycosylated, and containing hydrophilic domains [39]. The coronavirus envelope proteins contain a hydrophobic transmembrane domain and a C‐terminal domain containing motifs for glycosylation and palmitoylation [40]. Detailed crystallographic and binding studies will be required to establish whether SARS‐CoV‐2 envelope protein directly interacts with the TLR4/MD2 complex.

The reason for the increase in IL‐8 secretion in response to the combination of envelope protein and anti‐TLR4 compared to envelope protein plus control IgG is unknown. This could be due to trace impurities in the antibody acting in synergy with envelope protein to induce IL‐8 expression, although it seems unlikely, since this effect was not seen with other cytokines.

We found that SARS‐CoV‐2 spike protein did not induce the secretion of any of the measured cytokines from human AMs. This replicates what has been found in studies of murine BMDMs, human MDM [19], human PBMCs [15] and monocyte‐derived dendritic cells [41, 42]. On the other hand, other researchers have found THP‐1 and MDMs to be responsive to spike protein via TLR2 [11, 12]. This variability may be the result of reagent contamination [18], differences in experimental design or reagents used, or perhaps may reflect a true variability of response according to cell type. Indeed, tissue resident AMs are well described to be less pro‐inflammatory than monocytes or macrophages of monocyte origin [43, 44].

“Inflammaging” is characterized by higher basal circulating cytokines and altered responsiveness of myeloid lineage cells [45, 46]. We observed that AMs from donors over the age of 70 years had higher IL‐6, IL‐10, and IL‐1β secretion than younger donors following stimulation with SARS‐CoV‐2 envelope protein. To our knowledge, this is the first evidence of age affecting human AM cytokine responses. There are multiple possible mechanisms that could explain this difference. Stout‐Delgado et al. observed higher inflammasome activation after bleomycin stimulation in LPS‐primed AMs from aged mice [47]. Increased baseline inflammasome activation resulting from higher mitochondrial ROS or DNA has been proposed as an explanation for hyperinflammation in older patients with COVID‐19 [47, 48]. IL‐1β potentiates the release of IL‐6 and TNFα from SARS‐CoV‐2 infected monocytes, so its release could amplify the secretion of other cytokines [30]. SARS‐CoV‐2 envelope protein may activate the NLRP3 inflammasome through its function as a cation channel and TLR4 ligand. The second possible mechanism is differing baseline AM polarisation. Canan et al. reported that lung macrophages from aged mice had an activated phenotype [49]. Boe et al. found that AMs from aged mice had higher cell surface expression of TLR4– a third possible mechanism [50]. However, Droemann et al. found no difference in expression of cell surface TLR2 and TLR4 between AMs from young and elderly non‐smokers [51], although both their “young” and “old” groups were younger than those in our study and therefore may not be comparable. A fourth possible mechanism relates to the ontogeny of AMs. AMs are derived from the foetal yolk sac and are phenotypically distinct from MDMs. Over time, after repeated pulmonary immunological insults, these native AMs can be replaced by monocytes that originate from the bone marrow. These AMs of bone marrow origin make up a higher proportion of total AMs in the elderly and may have a distinct, pro‐inflammatory phenotype [43]. Although other research suggests that the lung microenvironment rather than the ontogeny of AMs dictates their behaviour [52]. However, ageing affects monocyte metabolism [53], which may have downstream effects on cellular responses to infection such as cytokine production, in particular IL‐1β [54]. Whatever the mechanism, the augmented cytokine responses in older donors may be relevant in explaining the worse disease trajectories older patients follow.

We also observed lower cytokine responses in AMs from active smokers following stimulation with envelope protein, when compared with never or ex‐smokers. Both groups had the same mean age. Our own laboratory has previously reported impaired TNFα, IFNγ and IL‐1β secretion in smokers' AMs, and observed diminished energetics in smokers' AMs responding to Mtb [25, 55]. This new finding concurs with that prior research and paints a picture of impaired pulmonary cytokine responses in smokers during SARS‐CoV‐2 infection. It is perhaps surprising that smoking has not been found to be a strong risk factor for severity in COVID‐19, unlike in influenza [19, 21, 22]. One speculative explanation is that the increased risk of severe disease attributable to chronic lung disease in smokers is counterbalanced by a protective effect—the blunted cytokine response of smokers AMs responding to SARS‐CoV‐2 envelope protein that we observed.

In addition to insights about COVID‐19 immunopathology, there are several possible practical applications of this research. One is the development of treatments: If AMs are a primary source of proinflammatory cytokines and chemokines in the lungs of COVID‐19 patients, then targeting AMs directly—perhaps through inhaled therapies—may reduce COVID‐19 immunopathology. Furthermore, we have shown that the envelope protein, and not the spike protein, provokes a cytokine response from human AMs. It follows that antibodies (therapeutic or vaccine induced) targeting the envelope protein might diminish this cytokine response. The envelope protein is highly genetically conserved and more stable than the spike protein [56]. Antibodies against the SARS‐CoV‐2 envelope protein can develop in response to natural infection [57]. Despite this, research on this topic has been limited to computational analyses of envelope protein epitopes [58, 59, 60, 61, 62] and one murine study of an experimental DNA‐based vaccine containing the envelope protein gene. The vaccine induced T and B cell immunity and prevented lung pathology following an inhalational challenge with SARS‐CoV‐2 [63]. The inclusion of the envelope protein as an antigen in COVID‐19 vaccines, alongside the spike protein, may improve their efficacy in preventing lung pathology in the elderly. Many vaccine adjuvants such as aluminium salts act through NLRP3 inflammasome activation [64]. The SARS‐CoV‐2 envelope protein forms cation channels in the host endoplasmic reticulum membrane that result in NLRP3 inflammasome activation [32, 33, 34, 35, 36]. Therefore, pairing the envelope protein with the spike protein in COVID‐19 vaccines may not only induce adaptive T cell responses (or perhaps anti‐envelope protein antibodies that could block its binding with TLR4) but also act as an adjuvant.

There are several limitations with this research. The scarcity of AM donors combined with inter‐donor variability and low cell counts manifests in several ways in this research. It is possible that our experiments are underpowered, and that the addition of donors would reveal that the effects of LPS are also dependent on age or smoking status. It is also possible that differences in cytokine secretion between younger and older donors, or smokers and never/ex‐smokers might be revealed using lower concentrations of LPS or other stimuli. In addition, we cannot fully rule out a role for trace bacterial material playing a role in the cytokine‐inducing activity of recombinant envelope protein. Our experiment showing that this activity is abrogated by protease digestion indicates that it is not due to the presence of non‐protein TLR agonists such as endotoxin and lipoteichoic acid. In support of a role for envelope protein as a TLR agonist, Xu et al. showed that ectopic expression of envelope protein (from a plasmid) in airway epithelial cells induced expression of proinflammatory cytokines similarly to the recombinant protein [37]. The degree to which experiments using SARS‐CoV‐2 antigens are relevant to natural infection is difficult to gauge. However, support for the relevance of envelope protein in SARS‐CoV‐2 pathogenesis comes from the findings that envelope protein alone causes lung inflammation in mice when inhaled, that an experimental envelope protein vaccine reduced lung pathology in mice, and that circulating envelope protein levels correlate with COVID‐19 severity in patients [9, 16, 63]. In vivo models of SARS‐CoV‐2 infection would also be better placed to examine the downstream consequences of AM responses to envelope protein on immune cell recruitment, as well as examining these responses in aged mice.

## 5 Conclusion

In SARS‐CoV‐2 infection, AMs of older adults might produce significantly more pro‐inflammatory cytokines following the recognition of SARS‐CoV‐2 envelope protein by TLR2 and TLR4. This effect may partly explain the worse clinical trajectories followed by these older patients. The inclusion of envelope protein as an antigen in COVID‐19 vaccines alongside spike protein may improve their efficacy in reducing pathology in older patients.

## Conflicts of Interest

The authors declare no conflicts of interest.

## Supporting information


**Data S1.** Supporting Information.

## Data Availability

The data that support the findings of this study are available on request from the corresponding author. The data are not publicly available due to privacy or ethical restrictions.
