# Supplementary material for: Human Alveolar Macrophages Detect SARS‐CoV‐2 Envelope Protein Through TLR2 and TLR4 and Secrete Cytokines in Response
Source: Immunology. 2025 May 4;175(3):391–401. doi: 10.1111/imm.13922 (PMC12130671; doi:10.1111/imm.13922)
Supplement: Supplementary file 1 — Data S1. Supporting Information. [file IMM-175-391-s001.docx]

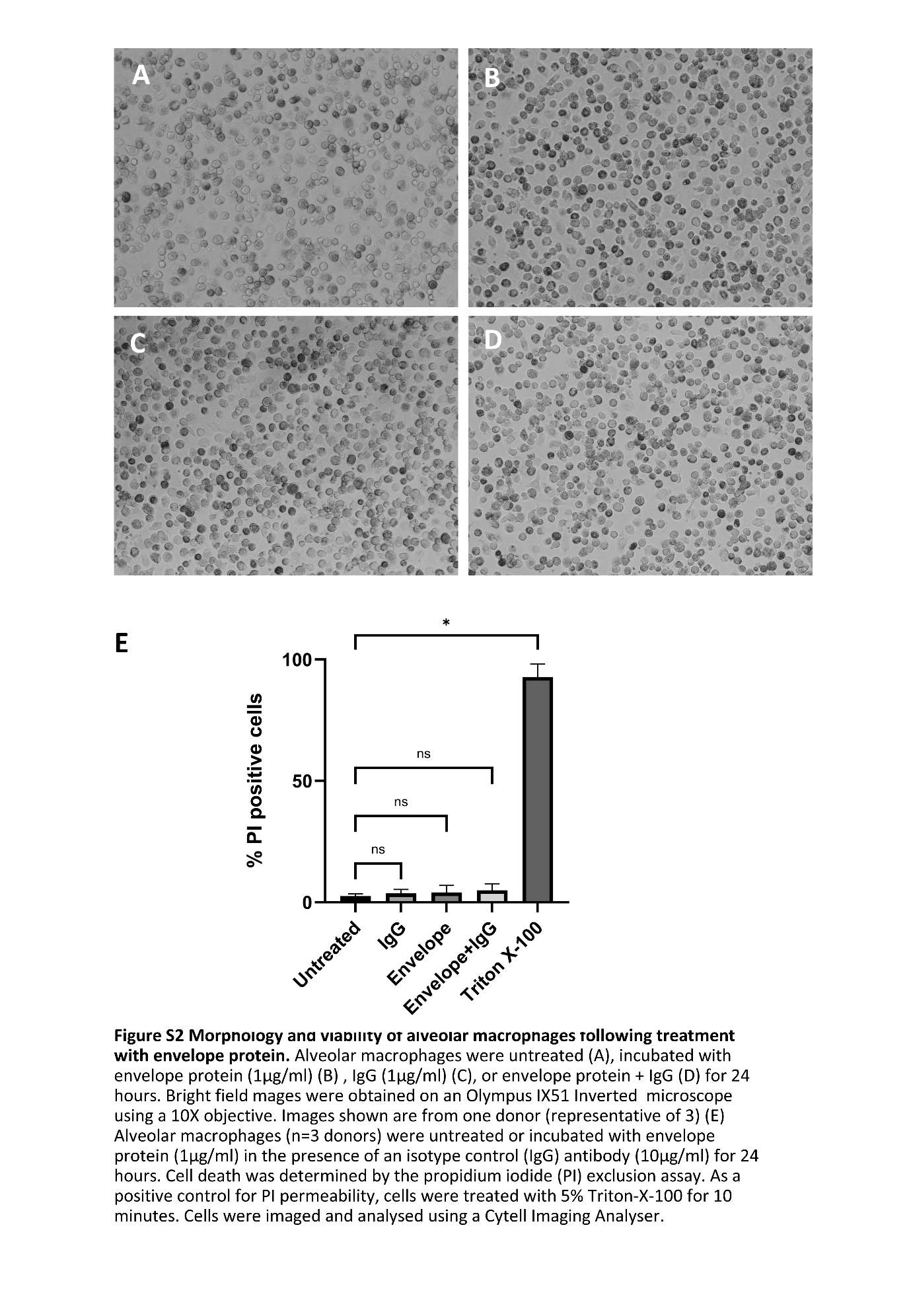


**Figure S1 Morphology and viability of alveolar macrophages following treatment with envelope protein.** Alveolar macrophages were (A) untreated, incubated with (B) envelope protein (1µg/ml), (C) IgG (10µg/ml), or (D) envelope protein + IgG for 24 hours. Bright field images were obtained on an Olympus IX51 Inverted microscope using a 10X objective. Images shown are from one donor (representative of 3). (E) Alveolar macrophages (n=3 donors) were untreated or incubated with envelope protein (1µg/ml) in the presence of an isotype control (IgG) antibody (10µg/ml) for 24 hours. Cell death was determined by the propidium iodide (PI) exclusion assay. As a positive control for PI permeability, cells were treated with 5% Triton-X-100 for 10 minutes. Cells were imaged and analysed using a Cytell Imaging Analyser.


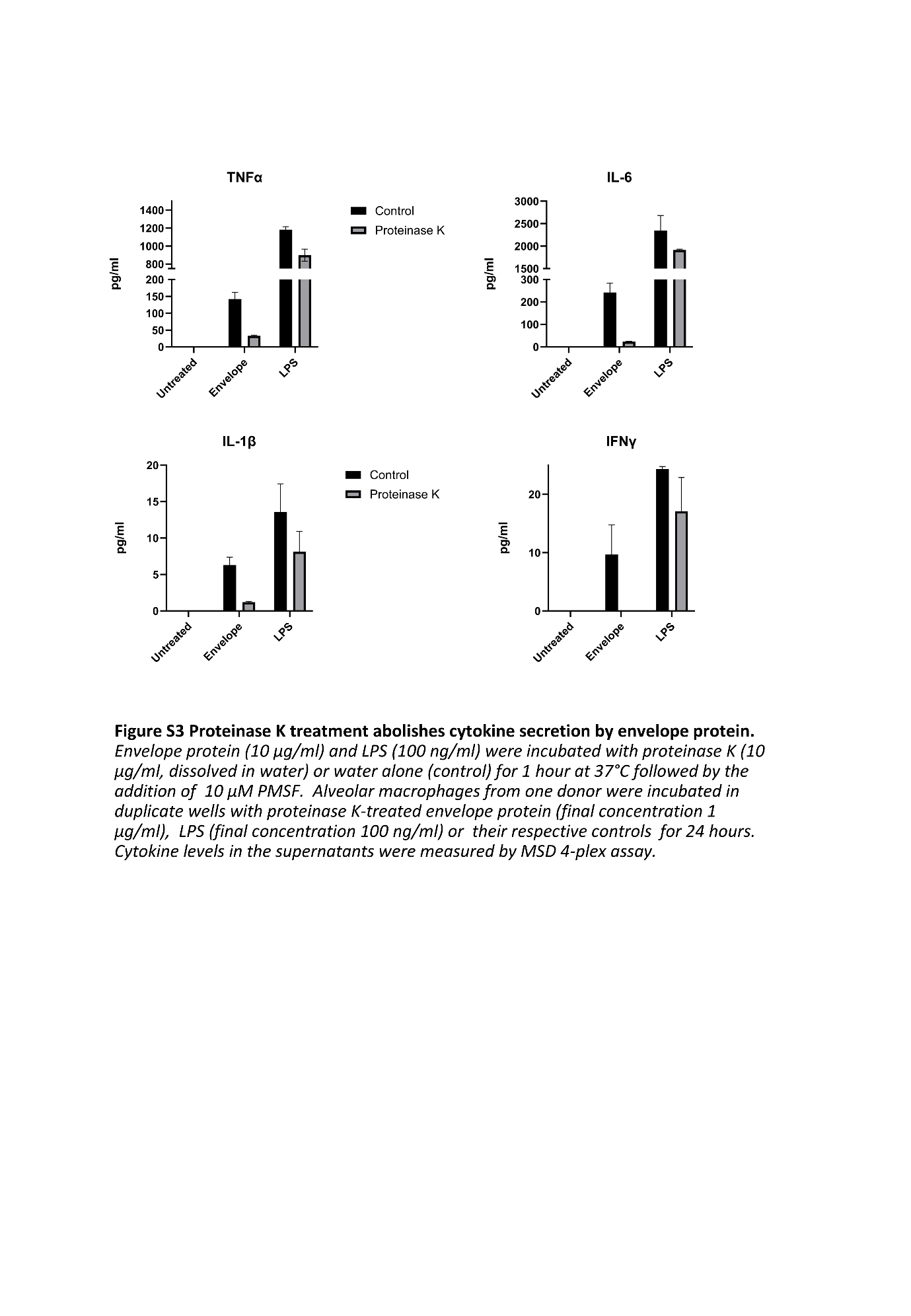


**Figure S2 Proteinase K treatment abolishes cytokine secretion by envelope protein.** Envelope protein (10 µg/ml) and LPS (100 ng/ml) were incubated with proteinase K (10 µg/ml, dissolved in water) or water alone (control) for 1 hour at 37°C followed by the addition of 10 µM PMSF. Alveolar macrophages from one donor were incubated in duplicate wells with proteinase K-treated envelope protein (final concentration 1 µg/ml), LPS (final concentration 100 ng/ml) or their respective controls for 24 hours. Cytokine levels in the supernatants were measured by MSD 4-plex assay.


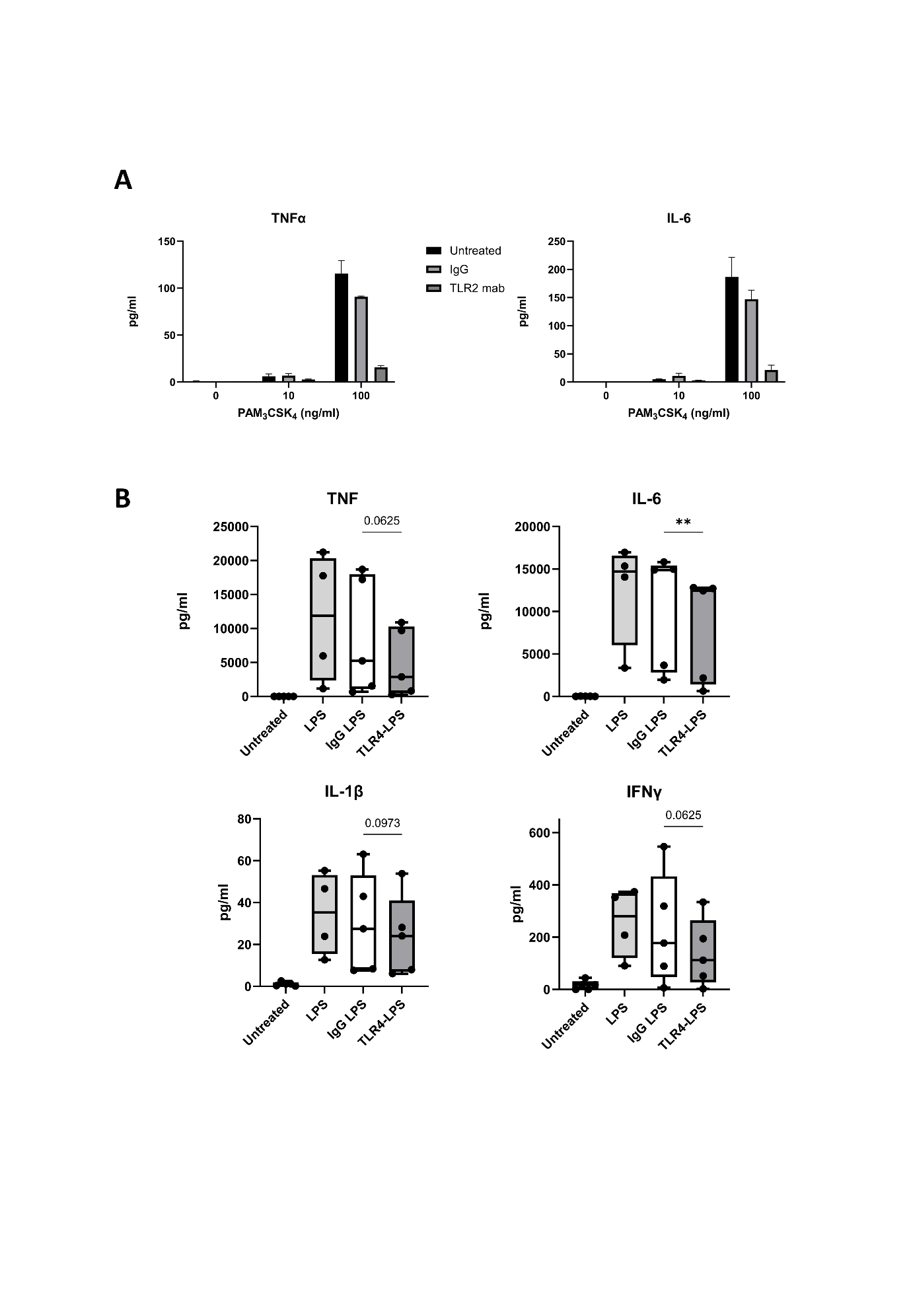


**Figure S3 Validation of TLR2 and TLR4 neutralising antibodies:**  Alveolar macrophages were (A) unstimulated or incubated with PAM_3_CSK_4_ at the indicated doses (n = 2) with or without control IgG or anti-TLR2 antibody (both a 10 µg/ml) or (B) LPS (100 ng/ml) in the presence or absence of IgG control antibody or neutralising TLR4 antibody (10 µg/ml) for 24 hours (n=5). Cytokine levels were measured in supernatants using a 4-plex MSD kit.
